# Evidence is not enough: health technology reassessment to de-implement low-value care

**DOI:** 10.1186/s12961-024-01249-w

**Published:** 2024-12-03

**Authors:** Sara Ingvarsson, Henna Hasson, Ulrica von Thiele Schwarz, Per Nilsen, Marta Roczniewska, Hanna Augustsson

**Affiliations:** 1 Centre for Psychiatry Research, Department of Clinical Neuroscience, Karolinska Institutet, Stockholm, Sweden; 2 Procome Research Group, Medical Management Centre, Department of Learning, Informatics, Management and Ethics, Karolinska Institutet, Stockholm, Sweden; 3 Unit for Implementation and Evaluation, Center for Epidemiology and Community Medicine (CES), Region Stockholm, Stockholm, Sweden; 4 School of Health, Care and Social Welfare, Mälardalen University, Västerås, Sweden; 5 Department of Health, Medical and Caring Sciences, Division of Public Health, Linköping University, Linköping, Sweden

**Keywords:** Low-value care, De-implementation, Health technology assessment, Health policy, Overuse, Disinvestment, Health care governance

## Abstract

**Background:**

The use of low-value care (LVC) is a persistent challenge in health care. Health technology reassessment (HTR) assesses the effects of technologies currently used in the health care system to guide optimal use of these technologies. Consequently, HTR holds promises for identifying and reducing, i.e., de-implementing, the use of LVC. There is limited research on how HTR is executed to support the de-implementation of LVC and whether and how HTR outcomes are translated into practical application. The aim of this study is to investigate how HTR is conducted to facilitate de-implementation of LVC and to investigate how the results of HTR are received and acted on in health care settings.

**Methods:**

This study is a qualitative interview study with representatives from health technology assessment agencies (*n* = 16) that support the regional health care organizations in Sweden and with representatives from the health care organizations (*n* = 7). Interviews were analysed with qualitative content analysis.

**Results:**

We identified three overarching categories for how HTR facilitates de-implementation of LVC and how the results are received and acted on in health care settings: (1) involving key stakeholders to facilitate de-implementation of LVC in identifying potential LVC practices, having criteria for accepting HTR targets, ascertaining high-quality reports and disseminating the reports; (2) actions taken by health care organization to de-implement LVC by priority setting and decision-making, networking between health care organizations and monitoring changes in the use of LVC practices; and (3) sustaining use of LVC by not questioning continued use, continued funding of LVC and by creating opinion against de-implementation.

**Conclusions:**

Evidence is not enough to achieve de-implementation of LVC. This has made health technology assessment agencies and health care organizations widen the scope of HTR to encompass strategies to facilitate de-implementation, including involving key stakeholders in the HTR process and taking actions to support de-implementation. Despite these efforts, there can still be resistance to de-implementation of LVC in passive forms, involving continued use of the practice and more active resistance such as continued funding and opinion-making opposing de-implementation. Knowledge from implementation and de-implementation research can offer guidance in how to support the execution phase of HTR.

**Supplementary Information:**

The online version contains supplementary material available at 10.1186/s12961-024-01249-w.

## Background

Reducing the extent of practices that provide no or little benefit to patients is a persistent challenge in health care. Use of low-value care (LVC), i.e. practices that are not clinically effective, have a poor risk–benefit profile or lack adequate evidence [1], is common. The prevalence of LVC ranges from 0.09 to 97.5 percent depending on the type of LVC and how it is measured [2]. Stopping or reducing the use of LVC requires deliberate processes that are often referred to as de-implementation, defined as a structured process to limit or completely stop the use of LVC. De-implementation has been studied in many different clinical fields, but has attracted limited attention from evidence and policy implementation scholars [3, 4].

Several overarching factors impact the use of LVC, spanning both inner and outer contexts, health care professionals’ knowledge and attitudes, patient expectations, and processes involved in managing LVC and de-implementation [3, 5]. Additionally, factors associated with the evidence supporting a practice, such as ambiguous guidelines, lack of awareness about a practice constituting LVC, challenges in applying guidelines to specific patient populations and distrust in the guideline sources, have been found to influence LVC use and de-implementation [3, 6, 7].

The scientific evidence on which guidelines are based is often produced through health technology assessment (HTA) performed by HTA agencies [8]. HTA is defined as “a multidisciplinary process that uses explicit methods to determine the value of a health technology at different points in its lifecycle”. The purpose is “to inform decision-making in order to promote an equitable, efficient and high-quality health system” [9]. HTA agencies typically engage in a comprehensive process encompassing identification, prioritization, assessment (including evidence analysis and evidence generation) and dissemination. Traditionally, HTA agencies have primarily focussed on evaluating the evidence of new technologies [10, 11]. However, recognizing the potential benefits, there is a growing recognition of employing similar methodologies to reassess already utilized technologies, a process termed health technology reassessment (HTR) [12].

HTR represents a parallel pursuit to HTA, focussing on evaluating the evidence supporting currently employed technologies. It constitutes a structured, evidence-based assessment of the clinical, social, ethical and economic effects of technologies (e.g. clinical practices and interventions) currently used in the health care system. The primary objective of HTR is to guide the optimal use of technologies when compared to available alternatives [13]. These technologies may include medications, diagnostic tests, devices, vaccines, or procedures. For this study, we will use the terms “technology” and “practice” interchangeably. Soril et al. [14] introduced a model outlining three sequential phases for HTR: (1) technology selection, which involves identification and prioritization of technologies to be reassessed, (2) decision, in which the evidence for the technology is being synthesized and used for developing a policy recommendation, and (3) execution, where the recommendations from the HTR are implemented, monitored and evaluated. The outcomes of HTR can vary, leading to recommendations such as increased use, no change in use, decreased use, or complete removal of the practice [12, 15] The latter two outcomes, in theory, are congruent with de-implementation. However, the HTR model is conceptual, and it remains to be investigated how HTR plays out in practice.

To limit LVC use, HTR recommendations needs to be implemented [15]. However, executing these recommendations poses challenges. Thus, HTR should be combined with de-implementation processes [12]. Several factors have been identified which facilitate de-implementation and/or disinvestment (i.e., withdrawal of funding from the technology following an HTR). These include ensuring a solid evidence base for the technology under reassessment, understanding the socio-political context, employing a structured HTR process, securing adequate resources and recognizing the roles of stakeholders [15]. Involving stakeholders with diverse roles and expertise in the HTR process can enhance the acceptance of the HTR outcomes and facilitate the de-implementation process [16]. It has also been suggested that strategies commonly employed for implementation purposes, such as champions, guidelines, education, reminders, audit and feedback, as well as incentives and policy, are vital in the HTR process to optimize use of a practice, so as to determine whether its use should be increased, unchanged, reduced or completely discontinued [12]. Despite the acknowledged challenges in realizing HTR outcomes, limited research has been conducted on how HTR is executed to support the de-implementation of LVC, as well as whether and how these outcomes are translated into practical application [17]. This study aims to contribute to this body of literature by offering empirical insights into the dynamics of this process.

### Aim

The aim of this study is to investigate how health technology reassessment is conducted to facilitate de-implementation of low-value care. Additionally, the study aims to investigate how the results of the health technology reassessment are received and acted on in health care settings.

## Methods

### Design

This study is a qualitative interview study including individual interviews with representatives from HTA agencies that support the regional health care organizations in Sweden and with representatives from the health care organizations. The study is reported according to the consolidated criteria for reporting qualitative research (COREQ) checklist [18].

### Setting

Sweden has a publicly funded and highly decentralized health care system in which the 21 regions (formerly known as county councils) are responsible for funding and providing health care to its citizens. The regions are politically governed by publicly elected representatives and with an administrative management that executes the region's activities, but the elected representatives have the ultimate responsibility. Both public and private health care providers operate in the publicly funded health care market [19].

Sweden operates with a comprehensive framework involving multiple government agencies dedicated to governing health care in different ways, including evaluation. Among these, the Swedish Agency for Health Technology Assessment and Assessment of Social Services stands as the national-level authority responsible for conducting independent assessments of methods used in health, medical and dental services. Alongside this central agency, Sweden maintains six intermediary HTA agencies, each focussed on specific health care regions or a cluster of regions. These intermediary agencies play a vital role by providing HTA services in collaboration with local health care organizations. To streamline processes and maintain consistency in assessment methodologies, these agencies function within a network that encompasses the national-level HTA agency and other national governance agencies. This network model is instrumental in preventing duplication of efforts while fostering a unified approach to HTA across the regional HTA agencies [20]. The primary task of these HTA agencies is to conduct both HTA and HTR. They accept inquiries from various stakeholders within health care organizations regarding the current evidence supporting specific practices. These inquiries could pertain to the introduction of new practices that organizations wish to implement or the reassessment of existing practices they are contemplating whether to de-implement [21].

### Participants and recruitment

The recruitment of participants to the study was conducted in two steps. In the first step, we identified representatives from each of the six HTA agencies from their web pages and invited them via e-mail to participate in the study. In some cases, the invited people recommended someone else in the organization that had more knowledge about the issue of interest. We also applied snowball sampling, whereby all informants were asked to suggest additional HTA representatives to interview. In total 20 individuals were invited in the first step, of whom 16 ultimately participated in the study (Table 1). Eleven of the informants were male. Reasons for not participating were not having knowledge about the issue of interest for the study (*n* = 3) and having retired (*n* = 1).

**Table 1:** Number of HTA agency representatives and health care organization representatives from each HTA agency

HTA agencies	HTA agency representatives	Health care organization representatives	
HTA centre—Västra Götalandsregionen	2	0	
HTA Sydöstra sjukvårdsregionen	3	1	
HTA Stockholm—Gotland	4	0	
HTA Norr	2	0	
Centre for Assessment of Medical Technology in Örebro	2	0	
HTA Syd/södra sjukvårdsregionen	3	6	
Total	16	7	

During the interviews we asked the informants if they had specific examples of HTR reports recommending decreased use or complete removal of a given practice. In the second step of recruitment, we identified the individuals that had submitted the question about these methods to the HTA agency. This information was provided either by snowball sampling from the first category of participants or in the HTR reports. The identified individuals were invited via email to participate in the study. In total 15 individuals representing thirteen HTR reports, were invited. Two individuals declined due to lack of time. One had changed jobs and did not feel knowledgeable enough about the topic, and five individuals did not respond to the invitation despite reminders. Seven informants, of which six were male, participated in the study (Table 1). They represented four different health care regions (related to reports from two HTA agencies) and had different roles in the health care system, either representing the regional health care organization or health care providers (then as, for example, hospital/clinic managers). The informants were involved in five HTR reports which had all resulted in an outcome recommending decreased use or complete discontinuation. The reports concerned surgical procedures, non-surgical procedures and behavioural health interventions. Thus, we included two categories of participants in the study: (1) HTA agency representatives and (2) health care organization representatives submitting questions to the HTA agencies that had resulted in reports that recommended that a given method should not be used. It should be noted that some of the HTA agency representatives worked within the health care organizations and thereby also provided information from that perspective in the interviews. All participants provided their informed consent prior to the interviews. The study has been approved by the Swedish Ethical Review Authority (reference number: 2019:02467).

### Data collection

Data was collected using semi-structured interviews. We used distinct interview guides tailored for each informant category (see additional file 1). These were informed by previous research on de-implementation of LVC (e.g. 3, 4) but were not developed based on a specific theory or framework. The interview guide designed for HTA agency representatives encompassed questions on their perspectives on LVC, their perceived and others’ potential roles in the de-implementation process, and the challenges associated with de-implementation. The interview guide intended for health care organization representatives included questions regarding the rationale behind requesting the HTR, how they managed the outcomes of the HTR, any resultant changes in practice, and their perceptions of the responsible party for ensuring the use of the results.

The interviews with HTA agency representatives were conducted by a research assistant holding a fil. Lic. degree, whereas interviews with health care organization representatives were conducted by two of the authors (S.I. and H.A.), both possessing PhD qualifications. All interviews were conducted via Zoom. The interviewers, all female, were trained in interview technique and had previous experience of conducting interviews. The two PhD holders had extensive expertise in research concerning the use and management of LVC, while the research assistant had limited prior exposure to the topic. None of the interviewers had any pre-existing relationships with the informants.

### Analysis

All interviews were transcribed verbatim and analysed using conventional qualitative content analysis (i.e. where categories are inductively derived from the text data) [22]. Interviews from both participant groups were analysed together. All transcribed interviews were initially read in their entirety to acquire a comprehensive overview of the content. Subsequently, line-by-line coding of the transcripts was executed using NVivo software. The coding process was carried out by SI, encompassing the initial inductive categorization of the data into categories and subcategories. Categories and sub-categories were subjected to continuous discussion among all authors and subsequent revisions were implemented based on these collaborative discussions.

## Results

**Fig. 1:**
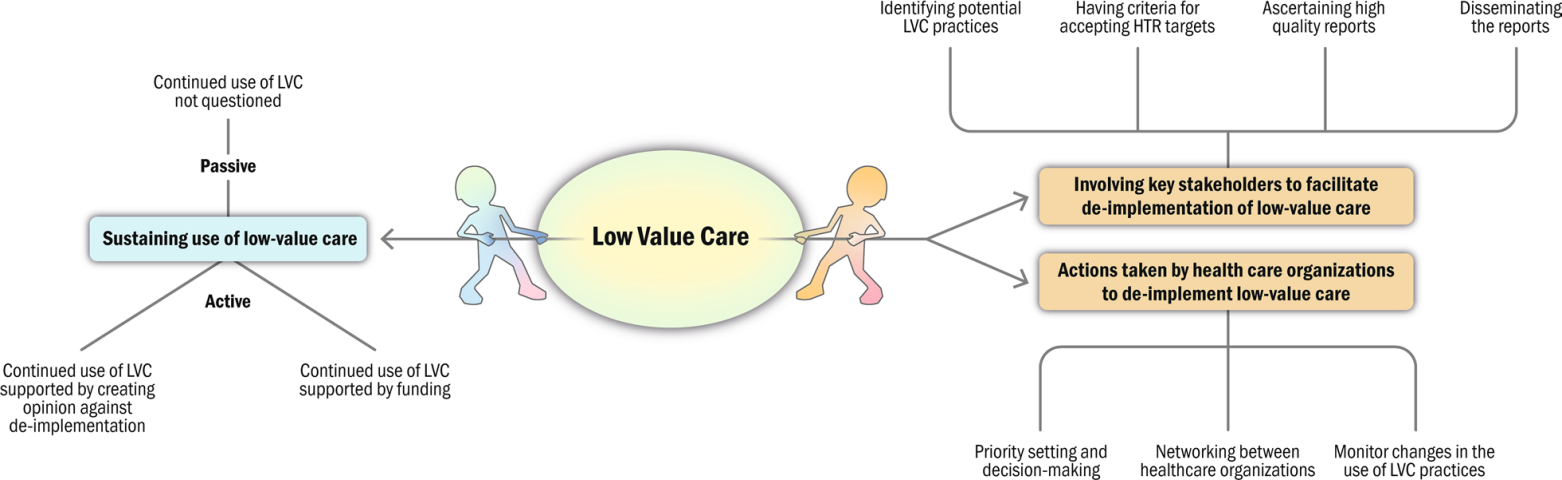
Categories and their sub-categories on how health technology reassessment is conducted to facilitate de-implementation of low-value care

The results from both participant categories, HTA agency representatives and health care organization representatives, are collectively presented. When pertinent, distinctions in the text specify the origin of the results from each participant category, alongside quotes. Three overarching categories were identified, outlining how HTR contributes to facilitating the de-implementation of LVC and how the results are received and acted on in health care settings: involving key stakeholders in the HTR process to facilitate de-implementation of LVC, actions taken by health care organization to support de-implementation of LVC, and sustaining use of LVC. The categories and their sub-categories are presented in Fig. 1.

### Involving key stakeholders in the HTR process to facilitate de-implementation of LVC

The involvement of key stakeholders in the HTR process was described as a way to facilitate de-implementation of LVC. The involvement was achieved in various ways and in different phases of the HTR process as illustrated within the four subcategories of identifying potential LVC practices, having criteria for accepting HTR targets, ascertaining high-quality reports and disseminating the reports. Not all types of involvement were pursued for all HTR processes.

#### Identifying potential LVC practices

Anyone within a health care organization served by a specific HTA agency could nominate a practice for HTR. This included health care professionals, managers, administrators and politicians. Nominations were described as mainly focussing on new technologies to be implemented, but there were examples of nominations of reassessments of existing practices where de-implementation could be relevant.

The health care organization representatives emphasized that not all de-implementation was done through HTR. Some practices were de-implemented simply by learning new, better practices. New research findings discussed among the health care professionals could sometimes be enough to decide to stop using a practice. If the discussion concerning the evidence for an existing practice was inconclusive, professionals would nominate the practice for an HTR, implying that the HTR process was used for more difficult questions.

#### Having criteria for accepting HTR targets

The criteria for accepting to conduct an HTR differed between the HTA agencies. Some only required that the question was relevant, whereas others also included a criterion that there should be local support for the HTR both during the process of reviewing the literature and for taking actions, e.g. de-implementation, based on the results of the assessment. Managers were expected to allocate personnel to participate in the relevant literature review processes and to be committed to making changes according to the results of the assessment. An illustrative quote here is as follows:

*For us to initiate an HTA process, we must have a commitment from the head of operations or clinic manager. Both that the individuals engaged as experts in the HTA working group have allocated time for it, but also that the manager is prepared to implement changes if the outcome indicates that changes are necessary. So, only when we have a written commitment from the manager regarding these two aspects do we begin (HTA representative 16).*

#### Ascertaining high-quality reports

The informants emphasized the importance of ensuring a rigorous process and a convincing report to minimize the risk of the results being questioned. To make sure that the process resulted in reports of high quality, the HTA agencies sought to involve people who had expertise concerning the practice to be evaluated, the research methodology for systematic reviews and health economics evaluations. This was supported by the health care organization representatives who described how information from the reports was sometimes disseminated by health care organization representatives involved in the HTR already during the process:

*They (clinic representatives participating in creating the HTR report) report continuously during the process so when the final report is presented, the findings are not new to us (health care organization representative 22).*

Another strategy to increase the likelihood of the report being accepted and the results being implemented was to include qualitative data in the report. Representatives described that there could be benefits from a practice that were not captured solely by scientific literature. In one case the report also included results from interviews with the health care professionals using the practice to capture the clinical experience of using the method to further strengthen the conclusions.

One of the participants expressed it like this:

*What we were mainly interested in was the application of the practice. In a living environment, not in a research environment. To do so, you need to both do interviews and go through the scientific publications (health care organization representative 19).*

#### Disseminating the reports

There was substantial variation in how the informants described the dissemination of the results of the HTA. Some HTA agencies took a rather passive approach and only reported the results to the person who nominated the practice to the HTA agency, with the report attached.

*We try to email the report to the person who posed the original question and to other people who may be directly involved or may be concerned in it, so they receive the information (HTA representative 3).*

Others took a more active approach and informed additional key stakeholders with an interest in the issue, such as medical societies or managers within organizations that would benefit from the report. This dissemination was still mainly done by e-mailing key stakeholders the report, with a message that the HTA agency thought that recipients would find the conclusions relevant. Another dissemination approach was to present the results to health care professionals currently using the practice. This was sometimes done by a manager, a staff member or an HTA agency representative. In connection to informing about the conclusions from the HTR, the professionals could discuss how to proceed with the conclusions and how to de-implement the practice in question. Having an assessment of the evidence was helpful in convincing the professionals that the practice was no longer useful and should be de-implemented:

*The managers said that they often referred to the report when they discussed the use of this practice (health care organization representative 18).*

Finally, some respondents reported that their region had a more comprehensive dissemination process, which included reporting to regional councils who made prioritizations within their health care organization on whether to de-implement a practice based on the HTR. This was part of a joint process between the HTA agencies and the health care organizations wherein the HTA agencies were invited to present their report verbally, including their conclusions that a given practice should be de-implemented:

*We have an assembly called the Program and Prioritization Council. They are the ones who make decisions in the region. There, those of us who have conducted these investigations are usually invited to present the findings (HTA representative 2).*

### Actions taken by health care organizations to support de-implementation of LVC

The health care organizations, including health care providers, conducted different actions to support de-implementation. The different actions can be illustrated within three subcategories: networking between health care organizations, priority setting and decision-making, and monitoring use of LVC practices.

#### Networking between health care organizations

Some of the health care organizations, mainly between regions, collaborated on de-implementing LVC. For instance, all regions served by the same HTA agency could combine forces and nominate a practice for HTR. They also collaborated on making similar priorities and tracking the use of a specific practice with the aim of achieving equal care across regions. Furthermore, they could observe the success or failure of de-implementation efforts in other regions and, based on those observations, decide on how to de-implement within their own region. Finally, involved organizations could initiate collaborations to conduct research on practices that had not yet received support from adequate evidence due to limited studies, but which were ranked as suitable for further studies.

All HTA agencies were also part of a national HTA network wherein they followed each other’s work and shared information on HTA/HTR reports to facilitate dissemination of results to health care organizations in other regions. The network was also mainly a forum to share reports and make sure that agencies did not investigate the same practice but could also result in joint efforts to inform key stakeholders of one of the agencies’ reports.

#### Priority setting and decision-making

Some of the informants described a defined and structured way of using the HTR reports for decision-making and priority setting. The reports were presented in one or several forums, e.g., regional health care management groups or method and prioritization councils tasked with forming materials and statements concerning both implementation and de-implementation of practices in health care. Depending on the type of practice, the decision-making forums could range from local managers to regional politicians. The discussion resulted in priority setting for the practices investigated in the HTA report. Some of the organizations used a similar grading as the Swedish National Board of Health and Welfare for prioritizing the evidence, ranging from one to ten, where ten was the least favourable level of evidence. The scale also included the label “Research and Development” for practices that lacked evidence and should be targets for research to assess their evidence for effectiveness. The label “Do not do” was used for practices that were advised against, i.e. LVC. Within these forums, a decision could be made to de-implement a certain practice with the intention of influencing the entire health care organization. One of the participants described this process in this way:

*It is important that these signals are communicated so that we do not differentiate, regardless of whether it is politicians or us making decisions, that we adhere to the evidence. That is what we aim to achieve (health care organization representative 20).*

When decisions were made throughout the different decision forums, the decisions were communicated to the clinics affected by the decision, and they would also make a formal decision to stop using a practice:

*We presented the report at our clinic’s meeting. I don’t remember who did this, this time, but we always have a representative from our clinic for these types of projects. Our representative reports about the findings during the review process, which means that once the report is finished, we are already aware of the results and can then de-implement according to the results (health care organization representative 22).*

#### Monitoring use of LVC practices

Following an HTR report recommending de-implementation, some of the health care organization representatives reported that they monitored key indicators related to the use of the LVC practice. This was perceived as a way of influencing health care professionals to limit their use of the practice and to put pressure on managers to focus on de-implementation. The results would then be presented both to the professionals and managers:

*We do this by modifying the documents in our management system, specifically the care guidelines and procedures. We incorporate the new approach into them, and then we continuously monitor the effects of that change (HTA agency representative 17)*

*One thing is that we regularly follow up, as in this case where we have done follow-up twice. Perhaps we should follow up a third time. It’s a way to put pressure on the managers (health care organization representative 18).*

### Sustaining use of low-value care

There were also examples of resistance to de-implementation. These actions pertain to three analytical subcategories: continued use not questioned, continued funding, and creating opinion against de-implementation.

#### Continued use not questioned

This action involved no strong reactions to the HTR, but simply continuing to use the practice that had been identified as LVC in the HTR. Some of the informants reported that sometimes health care professionals and managers did not agree with the HTR results and thus did not make decisions or efforts to de-implement the practice. Examples of reasons provided for this were that professionals had worked with the practice for a long time and had a positive experience of the practice, or that managers had experience of offering clinicians the opportunity to use a specific practice which helped in recruitment of skilled professionals:

*They saw other aspects, such as being able to recruit personnel, and based their decision on soft data (health care organization representative 19).*

When these conflicting beliefs and incentives existed, HTR results or decisions to de-implement a practice could be ignored. These examples were rarely described in combination with rigorous processes for prioritization and decision-making.

#### Continued use of LVC supported by funding

Continued funding of the LVC practice enabled continued use and prevented de-implementation. For instance, one example of LVC was provided by an external private care provider and had been procured by the regional health care politicians and could thus only be de-implemented by discontinuing the procurement of the practice. However, the procurement was not discontinued, even though the HTR showed that the practice lacked a scientific evidence base. The health care organization representatives also described other examples of LVC that were continued because of financial reimbursement for those practices, which provided a strong incentive for continued use, especially for private care providers:

*Pulling funding is a great method to put an end to things, but they don’t dare to do that (HTA agency representative 2).*

#### Continued use of LVC supported by creating opinion against de-implementation

Different ways of creating opinion to prevent de-implementation of LVC following HTR were also sometimes described. These efforts could be driven by health care professionals that did not accept the HTR results, as well as through engaging patients that were satisfied with the practice. Their individual stories about perceived benefits of a practice constituted compelling support for continuing use of the practice:

*It is a company that has been procured for this. They are very active. They have their patients who have used this practice that advocate this method and who are very satisfied (health care organization representative 20).*

The debate concerning the results of the HTR reports sometimes moved from the internal decision-making forums to more public forums such as opinion columns in newspapers and discussions in social media. These forums were used by health care professionals, patients and private providers and politicians to influence the public opinion regarding given LVC practices. Journalists were critiqued by the representatives for sometimes being more interested in creating debate than examining the scientific evidence:

*People go to the media, and newspaper articles are written about how this extremely important treatment that has helped so many is going to be discontinued and how can such an idiotic decision be made. And the media is not slow to jump on that circus without thoroughly investigating (health care organization representative 23).*

Another way of creating opinion against de-implementation was to influence politicians. For some of the LVC examples, a political decision was required to de-implement the practice, e.g. when the LVC required discontinuation of procurements or withdrawal of funding of the practice. Examples were provided where politicians had been influenced by public opinion, despite that the HTR-report showing that the practice lacked a scientific evidence base regarding effectiveness. A potential reason provided for this was the political system in which politicians are active may not be oriented towards taking unpopular decisions, since this may influence politicians’ possibilities of being re-elected. The health care organization representatives described how the evidence base and the structured process of HTR was overruled by public opinion in such cases—influencing politicians’ decisions concerning the practice and resulting in a decision to continue funding the practice:

*The opposition demands answers from the governing majority regarding why they are doing this, and the governing majority does not dare to argue, even though there is a substantial background work with scientific basis. Instead, they yield to some form of opinion that actually does not exist (health care organization representative 23).*

## Discussion

This study has sought to investigate how HTR is conducted to facilitate de-implementation of LVC and how the results of the HTR are received and acted on in health care settings. The results show that providing an HTR report was not perceived as sufficient for reducing or discontinuing the use of LVC. We identified two main analytic categories that pertained to activities facilitating de-implementation: involving key stakeholders in the HTR process and actions taken by health care organizations to support de-implementation of LVC. There were examples of HTR that led to de-implementation of LVC; however, there were also examples of different actors resisting de-implementation even when strategies to facilitate de-implementation had been used, as illustrated by the third category (sustaining use of LVC).

**Fig. 2:**
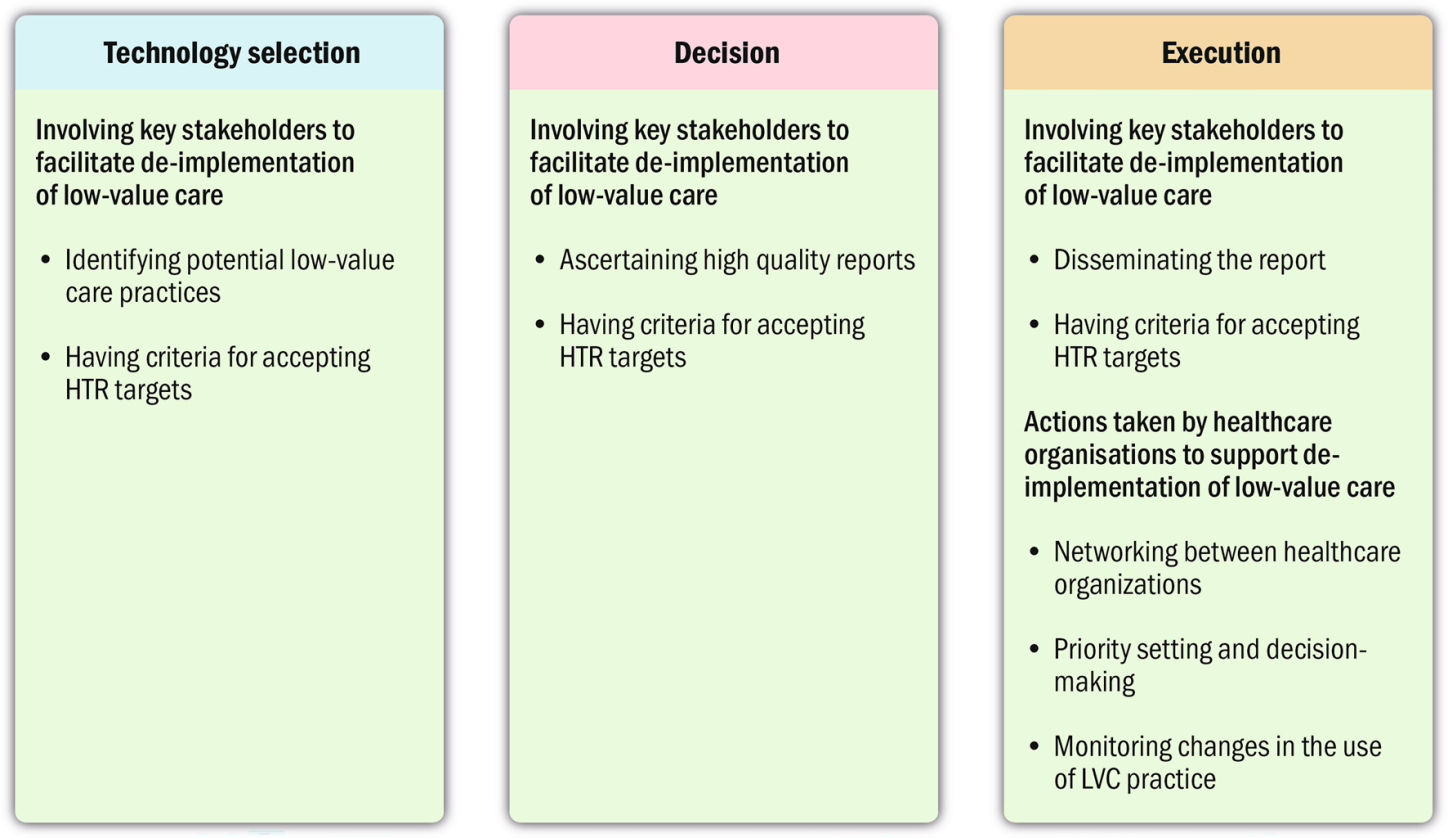
The different actions to facilitate de-implementation that the informants reported undertaking related to the different phases described in the conceptual model for HTR by Soril et al. [14]

The different actions to facilitate de-implementation that the informants reported undertaking can be related to the different phases described in the conceptual model for HTR by Soril et al. [14], as outlined in Fig. 2. The HTA agencies involved in the HTR lacked the formal decision authority to carry out the last step of the model, but could still take actions to facilitate de-implementation. In particular, they involved different stakeholders in the HTR process, which can help increase acceptability of the HTR and facilitate de-implementation [16]. The involvement of key stakeholders was described for all phases (technology selection, decision and execution). These findings support the emphasis on meaningful stakeholder engagement and ongoing knowledge exchange and utilization as foundational components that must exist in all phases of the HTR process to support acceptance of the HTR results. The actions taken by health care organizations to support de-implementation were all related to the final phase, execution, in which priority setting and decision-making, networking between organizations and monitoring of LVC use were reported as activities used to support de-implementation.

Some of the HTA agencies had a requirement of local support for the HTR, involving a commitment from the health care organizations to make changes if the HTR results identified a need for changes, before accepting to conduct the HTR. This approach can increase the likelihood of successful de-implementation, in that previous research has shown that there can be vested interests to retain LVC practices that can hinder de-implementation [3–5, 14]. Nevertheless, this entails the risk that LVC practices which are costly, ineffective or even harmful, but still supported by health care providers or patients, may be passed over for an HTR. Thus, there is an ethical imperative involved in HTR, to ensure use of practices that provide high quality of care for all patients [14]. However, HTR processes for practices that are supported by health care providers or patients likely need additional strategies to be de-implemented.

Despite efforts to facilitate de-implementation, providers sometimes resisted de-implementation. These efforts ranged from more passive resistance, through continued use, to more active resistance, including decisions to keep financing the practice and even efforts to create opinion against de-implementation. Both passive and active resistance underscore that evidence is not enough for de-implementation. This has also been shown in previous research, which has found that solely publishing guidelines recommending discontinuation of a practice is insufficient for de-implementation [23]. Passive resistance in the form of continued use was rarely described when the HTR results had been subject to structured processes of prioritization and decision-making. It is likely that when the HTR results were merely disseminated to the health care providers without discussion in prioritization and decision-making councils, it was possible to just continue using the practice. That is, more active efforts to prevent de-implementation were not necessary in these cases.

In some cases, even when thorough processes of prioritization and decision-making had been followed, efforts to create opinions against de-implementation succeeded in stopping the final decision to withdraw financing from the LVC practice in question. Several actors were described to be involved in such active resistance, including patients, health care providers, health care professionals, managers and politicians. The resistance to de-implementation was described as driven by different objectives: financial incentives for the health care providers, health benefits of current practice perceived by patients and/or health care professionals, the possibilities to attract skilled professionals for the managers and securing political support and votes for the politicians. Similar influences on LVC have been found in previous research [3, 5, 24]. These findings suggest that, in addition to the involvement of key stakeholders and support for HTR from health care organizations, existing motives to continue using LVC are multifactorial and need to be addressed to succeed with de-implementation of LVC.

In publicly funded health care systems, decisions about de-implementation may become a political issue. The informants described frustration with the regional politicians’ lack of understanding of how evidence is accumulated. However, there was also an understanding of the challenges of being a regional politician, since this is a position that is dependent on support from the public. Friedman et al. [25] suggest that social validity is one of three dimensions that should be considered when evaluating the scientific support for a practice, the others being the evidence and potential harm. Accounting for the social validity aspects makes it possible to weigh in the perception of the patients or the public, which can help to identify practices where the public opinion concerning a practice needs to be considered. This consideration could potentially be added to the HTR process to determine early in the process how the results may be received. If there is strong support for a given practice among health care professionals or patients, this may suggest that more extensive efforts are required to succeed with de-implementation.

The varied outcomes of how HTR results were put into practice in the examples provided in this study are in line with the assumption that execution is the most challenging phase in the HTR process. This phase requires additional effort. We support the suggestion by Esmail et al. [14] that HTR be complemented with knowledge from the implementation science field, which focusses on the systematic uptake of research findings and other evidence-based practices into routine practice [26]. This is essentially what the execution phase of HTR concerns. Implementation science applies numerous theories, models and frameworks to analyse factors acting as barriers and facilitators to implementation and evaluates the effectiveness of various strategies to support implementation. Similar theories, models and frameworks have also emerged for de-implementation applications [27–30]. Implementation science has increasingly accounted for LVC issues, since they obstruct implementation and use of practices based on evidence. Research is expanding on de-implementation strategies [3, 5], and also on which de-implementation strategies can be used to facilitate de-implementation as an output of HTR [31–33].

### Implications for research and practice

Despite growing understanding of the importance of de-implementation for LVC, there is still a knowledge gap concerning how resistance to de-implementation can be understood and what strategies can be added to the HTR process to overcome this. Particularly, there is a need for studies focussing specifically on how to address the main barriers associated with key stakeholders. These include the presence of patients with strong beliefs in a certain practice, the presence of health care professionals who have had positive experiences from using a specific practice, the needs of managers to recruit skilled professionals, the interests of health care providers who have a substantial part of their financing from using a specific practice, and the interests of politicians who depend on support from their constituents.

Related to implications for practice, it seems like both processes for involving key stakeholders and gaining organizational support for de-implementation are relevant efforts to enable de-implementation. However, such processes need to play out in combination with other efforts to understand and handle resistance to de-implementation.

### Methodological considerations

One principal strength of our study lay in the inclusion of representatives both for HTA agencies and health care, allowing for a more comprehensive understanding of how different phases of HTR relate to de-implementation of LVC. Furthermore, we interviewed representatives from six different agencies, yielding information on different ways of conducting HTR. Tracking the outcomes of specific HTR reports yielded information about different ways in which reports were received and acted on.

One principal limitation of our study is that we relied on the nominations from HTA agencies to identify HTR reports and thereby also to sample health care organization representative informants, resulting in a possibly skewed sample of healthcare representatives. This was further affected by the fact that only reports from two HTA agencies were represented among the health care organization representatives. There may be other relevant HTR cases which were not included in the study. Nevertheless, different types of LVC practices that had resulted in varied de-implementation outcomes (ranging from de-implementation to passive resistance to active resistance) were identified and included in the study.

The findings represent activities which the representatives reported as having been used within HTR to facilitate de-implementation. We have limited knowledge of how often and to what extent the reported activities were pursued. It is possible that the descriptions give a sense of more extensive work on facilitating de-implementation in the HTR process than what has taken place. However, it is also possible that respondents overlooked some activities. Moreover, we were not able to link different approaches to conducting the HTR to the de-implementation outcomes. This would require a larger sample of HTR cases.

The study has been conducted in Sweden that has a system of both national-level and regional-level HTA agencies responsible for HTR and a mostly publicly funded healthcare system in which regions are responsible for the funding and provision of healthcare to its citizens. Consequently, the findings may not be directly transferred to other healthcare systems. Nevertheless, we believe that identified strategies to facilitate de-implementation in the HTR process also apply also to other countries, which is supported by the overlapping process of how HTR was conducted in this study with the conceptual model for HTR proposed by Soril et al. [14].

## Conclusion

Evidence of lack of effectiveness is not enough to achieve de-implementation of LVC. This has made HTA agencies and health care organizations widen the scope of HTR to include specific strategies to facilitate de-implementation, including involving key stakeholders in the HTR process and taking actions within health care organizations to support de-implementation. In addition to evidence, other goals and values may influence whether a LVC practice is de-implemented or not following a HTR report, which may even result in resistance to de-implementation of LVC. This can involve passive efforts, involving continued use of the practice, but also more active resistance such as continued funding and opinion formation opposing de-implementation. Knowledge from implementation and de-implementation research can offer guidance in how to support the execution phase of HTR.

## Supplementary Information

Additional file 1.

Additional file 2.

Additional file 3.

## Data Availability

The datasets generated and/or analysed during the current study are not publicly available due to integrity of participants but are available from the corresponding author on reasonable request.

## Abbreviations

HTA: Health technology assessment
HTR: Health technology reassessment
LVC: Low-value care
