# Supplementary material for: Evidence is not enough: health technology reassessment to de-implement low-value care
Source: Health Res Policy Syst. 2024 Dec 3;22:159. doi: 10.1186/s12961-024-01249-w (PMC11613514; doi:10.1186/s12961-024-01249-w)
Supplement: Supplementary file 1 — Additional file 1. [file 12961_2024_1249_MOESM1_ESM.docx]

**Intervview guide Regional Studie**:

**Introduction** – a brief background on what the interview will be about and the definition of terms:

"We are conducting a study that focuses on the governance of the phase-out of low-value care. By low-value care, I mean healthcare services that have little value for the patient and the healthcare system due to a lack of evidence of effectiveness or even potential harm to the patient, or because the costs outweigh the benefits. Common examples of low-value care include unnecessary antibiotic prescriptions or medications that are not suitable for individuals over 65, unnecessary X-rays, and unnecessary lab tests. The purpose of the study is to understand more about how phase-out decisions are made, the activities undertaken to influence phase-out, and the challenges related to reducing low-value care.

As part of this project, we have interviewed representatives from regional method councils/HTA organizations to learn about their approach to phase-out. In one such interview, your inquiry regarding xxxxxxxxx was mentioned. In this interview, we are interested in learning more about what happened after the report on xxxxxxx was produced.

**Consent & Confidentiality** – a brief reminder:

“With your consent, the interview will be digitally recorded, and transcribed transcripts will be used for data analysis. No names or other information that could identify the interviewees will be included when presenting the study results.”

**Personal Questions**:

1. Can you briefly describe your role within the (actor’s organization/unit)?

**The Actor’s Work on Phase-Out**:

1. How did you come to pose the question to HTA Skåne?
2. Who was involved in formulating the question?
3. Based on the literature search, what did HTA (Skåne) conclude?
4. How have you utilized the results from the report?
5. What effects, if any, has the report had on the use of hyperbaric oxygen therapy for diabetic foot ulcers, radiation…
