# Supplementary material for: Evidence is not enough: health technology reassessment to de-implement low-value care
Source: Health Res Policy Syst. 2024 Dec 3;22:159. doi: 10.1186/s12961-024-01249-w (PMC11613514; doi:10.1186/s12961-024-01249-w)
Supplement: Supplementary file 2 — Additional file 2. [file 12961_2024_1249_MOESM2_ESM.docx]

# Interview guide – HTA representatives.

**Introduction – A Brief Background on the Interview Topic and Definition of Terms**

*This interview will focus on the role that you, as part of the [insert HTA agency], play in the governance of de-implementation of low-value care. By ‘low-value care,’ I refer to healthcare interventions that provide little value to patients and the healthcare system. Common examples of low-value care include non-indicated prescription of antibiotics, or medications unsuitable for individuals over 65, unnecessary X-rays, and unnecessary lab tests. It can also occur when costs outweigh the benefits (i.e., lack of cost-effectiveness).*

*When I mention ‘care,’ I encompass a wide range of interventions, from medications to other forms of treatment, as well as activities such as lab tests and diagnostic methods.*

*In this study, we focus on regional actors who can influence de-implementation, meaning the reduction or complete cessation of low-value care practices. The purpose of this study is to gain insights into how regional actors perceive their own roles and responsibilities, the activities undertaken to impact de-implementation, and the challenges faced by regional actors in influencing the de-implementation of low-value care.*

**Current work on de-implementation**

1. Can you briefly describe your role at the [insert HTA agency]?
2. What experiences do you have in working with issues related to de-implementation of low-value care? Can you provide an example?
3. Who was involved in conducting the Health Technology Reassessment (HTR)? What happened after the HTR report was produced? How has it been disseminated/used?
4. Who should have reacted to the report in your region – who can influence?
5. What would have been needed for the results to have a greater impact on the de-implementation of the low-value care practice?
6. When we talk about de-implementation of low-value care, what do you think is included in that concept?
7. If you were to describe how your HTA agency works on issues related to de-implementation of low-value care, what activities do you engage in concretely?
8. Are there other things you do that indirectly affect the de-implementation of low-value care?
9. What challenges exist in your work related to de-implementation?

**View on the HTA agency´s role and responsibility – Regional level**

1. Do you see any challenges in governing de-implementation at the regional level? What could be improved?
2. Based on the regional governance level, how would you describe the role of the ”insert HTA agency” for the de-implementation of low-value care?
3. How do you perceive your responsibility regarding de-implementation pf low-value care? Do you have any responsibility?
4. In relation to the other things you do, how important is the de-implementation of low-value care in your work?

**View on Others’ Roles and Responsibilities – Regional Level**

1. (Sampling question) Which other actors at the regional level influence the de-implementation of low-value care? In what way?
2. Are there any examples of regions where you know they have worked on de-implementation?

**View on Others’ Roles and Responsibilities – National Level**

1. There are also various actors at the national level who could impact the de-implementation of low-value care. Is there anyone among them whom you think affects your work? How?
2. How do you perceive the governance of de-implementation at the national level?

**View on Professional Autonomy**

1. What role do you believe the profession plays in the de-implementation of low-value care?
2. How do you view their responsibility? What can be expected of them?
3. In the de-implementation work carried out by your HTA agency, how do you consider the profession’s autonomy and their ability to make independent decisions in patient care?
4. How does the profession impact your de-implementation work at the regional level?

**The Patient**

1. What role do patients and patient associations play in the de-implementation of low-value care?

**Challenges and Opportunities**

1. What do you think works particularly well in the governance of de-implementation?
2. How do you see the future of de-implementation governance? Will there be any changes?
3. Anything else you’d like to add?
4. (Sampling question) Is there anyone else in your organization with knowledge and experience of these issues whom we should meet?
